# Bis(2,6-dimethyl­pyridine-κ*N*)gold(I) tetra­chloridoaurate(III)

**DOI:** 10.1107/S1600536808027876

**Published:** 2008-09-06

**Authors:** Roya Ahmadi, Leila Dehghan, Vahid Amani, Hamid Reza Khavasi

**Affiliations:** a Islamic Azad University, Shahr-e-Rey Branch, Tehran, Iran; b Department of Chemistry, Shahid Beheshti University, Tehran 1983963113, Iran

## Abstract

In the cation of the title compound, [Au(C_7_H_9_N)_2_][AuCl_4_], the Au^I^ atom is two-coordinated in a linear arrangement by two N atoms from two 2,6-dimethyl­pyridine ligands. In the anion, the Au^III^ atom has a virtually square-planar coordination geometry. The Au atoms both are located on centers of inversion. The crystal structure involves inter­molecular C—H⋯Cl hydrogen bonds.

## Related literature

For related literature, see: Abbate *et al.* (2000 ▶); Adams & Strähle (1982 ▶); Ahmadi *et al.* (2008 ▶); Amani *et al.* (2008 ▶); Bjerne­mose *et al.* (2004 ▶); Hayoun *et al.* (2006 ▶); Hojjat Kashani *et al.* (2008 ▶); Hollis & Lippard (1983 ▶); McInnes *et al.* (1995 ▶); Yildirim *et al.* (2008 ▶).
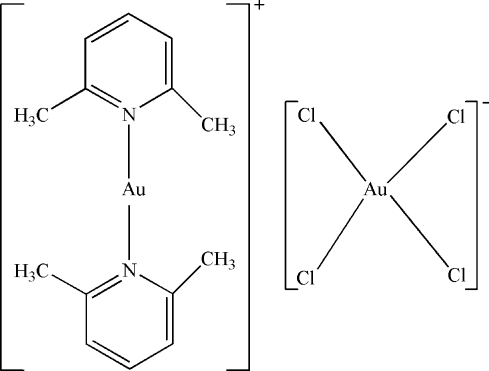


## Experimental

### 

#### Crystal data

[Au(C_7_H_9_N)_2_][AuCl_4_]
*M* *_r_* = 750.04
Monoclinic, 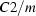

*a* = 17.773 (3) Å
*b* = 6.8395 (8) Å
*c* = 8.3728 (14) Å
β = 110.929 (12)°
*V* = 950.6 (3) Å^3^
*Z* = 2
Mo *K*α radiation
μ = 15.97 mm^−1^
*T* = 298 (2) K
0.20 × 0.12 × 0.08 mm

#### Data collection

Bruker SMART APEX CCD area-detector diffractometer
Absorption correction: multi-scan (*SADABS*; Sheldrick, 1996 ▶) *T*_min_ = 0.112, *T*_max_ = 0.275
5473 measured reflections
1384 independent reflections
1123 reflections with *I* > 2σ(*I*)
*R*_int_ = 0.092

#### Refinement

*R*[*F* ^2^ > 2σ(*F* ^2^)] = 0.044
*wR*(*F* ^2^) = 0.115
*S* = 1.20
1384 reflections
69 parameters
H-atom parameters constrained
Δρ_max_ = 1.76 e Å^−3^
Δρ_min_ = −2.1 e Å^−3^

### 

Data collection: *SMART* (Bruker, 2007 ▶); cell refinement: *SAINT* (Bruker, 2007 ▶); data reduction: *SAINT*; program(s) used to solve structure: *SHELXTL* (Sheldrick, 2008 ▶); program(s) used to refine structure: *SHELXTL*; molecular graphics: *SHELXTL*; software used to prepare material for publication: *WinGX* (Farrugia, 1999 ▶).

## Supplementary Material

Crystal structure: contains datablocks global, I. DOI: 10.1107/S1600536808027876/hy2150sup1.cif

Structure factors: contains datablocks I. DOI: 10.1107/S1600536808027876/hy2150Isup2.hkl

Additional supplementary materials:  crystallographic information; 3D view; checkCIF report

## Figures and Tables

**Table:** 

Au1—N1	2.030 (8)
Au2—Cl1	2.280 (3)
Au2—Cl2	2.286 (4)

**Table:** 

Cl1^i^—Au2—Cl2	90.05 (14)
Cl1—Au2—Cl2	89.95 (14)

Symmetry code: (i) 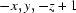.

**Table 2:** Hydrogen-bond geometry (Å, °)

*D*—H⋯*A*	*D*—H	H⋯*A*	*D*⋯*A*	*D*—H⋯*A*
C3—H3⋯Cl1^ii^	0.93	2.77	3.572 (14)	145

Symmetry code: (ii) 
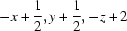
.

**Fig. 1.**
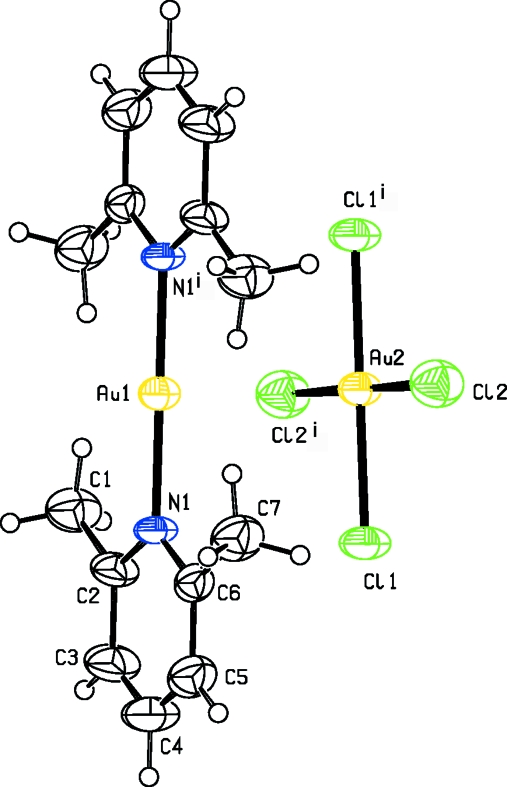
The molecular structure of the title compound. Displacement ellipsoids are drawn at the 40% probability level. [Symmetry code: (i) -x, y, 1 - z.]

## supplementary crystallographic information

### Comment

Recently, we reported the syntheses and crystal structures of [Au(dmphen)Cl_2_][AuCl_4_], (II), (Ahmadi *et al.*, 2008), [dmpyH][PtCl_6_], (III), (Amani *et al.*, 2008) and [H_2_DA18C6][AuCl_4_].2H_2_O, (IV), (Hojjat Kashani *et al.*, 2008) (dmphen = 4,7-diphenyl-1,10-phenanthroline; dmpyH = 2,6-dimethylpyridinium and H_2_DA18C6 = 1,10-diazonia-18-crown-6). Several Au^III^ complexes with formula [AuCl_2_*L*]*X*, such as [AuCl_2_(bipy)](BF_4_), (V), (McInnes *et al.*, 1995), [AuCl_2_(bipy)](NO_3_), (VI), (Bjernemose *et al.*, 2004), [AuCl_2_(bipy)][AuBr_4_], (VII), (Hayoun *et al.*, 2006), [AuCl_2_(dtbpy)][AuCl_4_].CH_3_CN, (VIII), (Yildirim *et al.*, 2008) and [AuCl_2_(phen)]Cl.H_2_O, (IX), (Abbate *et al.*, 2000) (bipy = 2,2'-bipyridine; dtbpy = 4,4'-ditertbutyl-2,2'-bipyridine; phen = 1,10-phenanthroline) have been synthesized and characterized by single-crystal X-ray diffraction methods. Two Au^III^ complexes with formula [AuCl_2_*L*_2_]*X*, [AuCl_2_(py)_2_][AuCl_4_], (*X*), and [AuCl_2_(py)_2_]Cl.H_2_O, (XI), (Adams & Strahle, 1982) (py = pyridine) and only one mixed-valence Au^I^–Au^III^ complex, [Au(terpy)Cl]_2_[AuCl_2_]_3_[AuCl_4_], (XII), (Hollis & Lippard, 1983) (terpy = 2,2',2''-terpyridine) have been synthesized and characterized by single-crystal X-ray diffraction methods. We report herein the synthesis and crystal structure of the title compound (I).

In the cation of the title compound (Fig. 1), the Au^I^ atom is two-coordinated in a linear arrangement by two N atoms from two 2,6-dimethylpyridine ligands. In the anion, the Au^III^ atom has a square-planar coordination geometry. The Au atoms each are located on an inversion center. In the cation, the Au—N bond length (Table 1) is in good agreement with the corresponding values in (*X*) and (XI) and in the anion, the Au—Cl bond lengths and angles (Table 1) are within a normal range, comparable with those in (II), (VIII) and (XII). In the crystal structure, intermolecular C—H···Cl hydrogen bonds are observed.

### Experimental

A solution of 2,6-dimethylpyridine (0.12 g, 1.09 mmol) in methanol (15 ml) was added to a solution of HAuCl_3_.3H_2_O, (0.37 g, 1.09 mmol) in acetonitrile (15 ml). The resulting yellow solution was stirred for 10 min at 313 K, and then it was left to evaporate slowly at room temperature. After one week, yellow block crystals of (I) were isolated (yield 75.8%, 0.31 g; m. p. 489 K).

### Refinement

All H atoms were positioned geometrically and refined as riding atoms, with C—H = 0.93 (aromatic) and 0.96 (methyl) Å and with *U*_iso_(H) = 1.2*U*_eq_(C). The highest residual electron density was found 0.90 Å from atom Au2 and the deepest hole 0.81 Å from atom Au2.

### Crystal data

**Table:**

[Au(C_7_H_9_N)_2_][AuCl_4_]	*F*(000) = 684
*M**_r_* = 750.04	*D*_x_ = 2.62 Mg m^−^^3^
Monoclinic, *C*2/*m*	Mo *K*α radiation, λ = 0.71073 Å
Hall symbol: -C 2y	Cell parameters from 971 reflections
*a* = 17.773 (3) Å	θ = 2.6–29.2°
*b* = 6.8395 (8) Å	µ = 15.97 mm^−^^1^
*c* = 8.3728 (14) Å	*T* = 298 K
β = 110.929 (12)°	Block, yellow
*V* = 950.6 (3) Å^3^	0.20 × 0.12 × 0.08 mm
*Z* = 2	

### Data collection

**Table:**

Bruker SMART APEX CCD area-detector diffractometer	1123 reflections with *I* > 2σ(*I*)
φ and ω scans	*R*_int_ = 0.092
Absorption correction: multi-scan (SADABS; Sheldrick, 1996)	θ_max_ = 29.2°, θ_min_ = 2.6°
*T*_min_ = 0.112, *T*_max_ = 0.275	*h* = −23→24
5473 measured reflections	*k* = −9→8
1384 independent reflections	*l* = −11→11

### Refinement

**Table:**

Refinement on *F*^2^	0 restraints
Least-squares matrix: full	H-atom parameters constrained
*R*[*F*^2^ > 2σ(*F*^2^)] = 0.044	*w* = 1/[σ^2^(*F*_o_^2^) + (0.0468*P*)^2^ + 5.4887*P*] where *P* = (*F*_o_^2^ + 2*F*_c_^2^)/3
*wR*(*F*^2^) = 0.115	(Δ/σ)_max_ = 0.004
*S* = 1.20	Δρ_max_ = 1.76 e Å^−^^3^
1384 reflections	Δρ_min_ = −2.1 e Å^−^^3^
69 parameters	

### Special details

**Table:**

Geometry. All e.s.d.'s (except the e.s.d. in the dihedral angle between two l.s. planes) are estimated using the full covariance matrix. The cell e.s.d.'s are taken into account individually in the estimation of e.s.d.'s in distances, angles and torsion angles; correlations between e.s.d.'s in cell parameters are only used when they are defined by crystal symmetry. An approximate (isotropic) treatment of cell e.s.d.'s is used for estimating e.s.d.'s involving l.s. planes.

### Fractional atomic coordinates and isotropic or equivalent isotropic displacement parameters (Å^2^)

**Table:**

	*x*	*y*	*z*	*U*_iso_*/*U*_eq_	
Au1	0	0.5	0.5	0.04157 (18)	
Au2	0	0	0.5	0.0484 (2)	
Cl1	0.13598 (17)	0	0.6416 (5)	0.0656 (9)	
Cl2	0.0196 (2)	0	0.2445 (5)	0.0726 (10)	
N1	0.1180 (5)	0.5	0.6531 (12)	0.044 (2)	
C1	0.0754 (8)	0.5	0.8993 (16)	0.068 (4)	
H1A	0.0803	0.6146	0.9684	0.081*	
H1B	0.0238	0.5	0.8084	0.081*	
C2	0.1398 (7)	0.5	0.8262 (15)	0.054 (3)	
C3	0.2206 (8)	0.5	0.9317 (17)	0.071 (4)	
H3	0.2353	0.5	1.05	0.085*	
C4	0.2774 (8)	0.5	0.859 (2)	0.079 (5)	
H4	0.3315	0.5	0.9288	0.095*	
C5	0.2568 (8)	0.5	0.684 (2)	0.065 (3)	
H5	0.2965	0.5	0.6361	0.078*	
C6	0.1763 (6)	0.5	0.5816 (15)	0.047 (2)	
C7	0.1497 (8)	0.5	0.3949 (19)	0.069 (4)	
H7A	0.1702	0.386	0.3574	0.083*	
H7B	0.0919	0.5	0.3473	0.083*	

### Atomic displacement parameters (Å^2^)

**Table:**

	*U*^11^	*U*^22^	*U*^33^	*U*^12^	*U*^13^	*U*^23^
Au1	0.0319 (3)	0.0531 (4)	0.0374 (3)	0	0.00961 (19)	0
Au2	0.0327 (3)	0.0502 (4)	0.0538 (3)	0	0.0053 (2)	0
Cl1	0.0327 (12)	0.071 (2)	0.077 (2)	0	−0.0006 (12)	0
Cl2	0.0641 (19)	0.090 (3)	0.0611 (18)	0	0.0195 (15)	0
N1	0.024 (3)	0.050 (5)	0.050 (5)	0	0.002 (3)	0
C1	0.060 (7)	0.097 (12)	0.045 (6)	0	0.017 (5)	0
C2	0.038 (5)	0.065 (8)	0.042 (5)	0	−0.006 (4)	0
C3	0.047 (6)	0.098 (12)	0.047 (6)	0	−0.008 (5)	0
C4	0.036 (6)	0.098 (13)	0.086 (10)	0	0.002 (6)	0
C5	0.045 (6)	0.075 (10)	0.078 (9)	0	0.026 (6)	0
C6	0.040 (5)	0.050 (6)	0.053 (6)	0	0.020 (4)	0
C7	0.057 (7)	0.088 (11)	0.074 (9)	0	0.037 (7)	0

### Geometric parameters (Å, °)

**Table:**

Au1—N1	2.030 (8)	C2—C3	1.392 (15)
Au1—N1^i^	2.030 (8)	C3—C4	1.35 (2)
Au2—Cl1^i^	2.280 (3)	C3—H3	0.93
Au2—Cl1	2.280 (3)	C4—C5	1.38 (2)
Au2—Cl2	2.286 (4)	C4—H4	0.93
Au2—Cl2^i^	2.286 (4)	C5—C6	1.381 (17)
N1—C2	1.360 (15)	C5—H5	0.93
N1—C6	1.370 (14)	C6—C7	1.463 (18)
C1—C2	1.479 (18)	C7—H7A	0.96
C1—H1A	0.96	C7—H7B	0.96
C1—H1B	0.96		
			
N1—Au1—N1^i^	180.0 (3)	C4—C3—C2	118.8 (12)
Cl1^i^—Au2—Cl1	180.00 (8)	C4—C3—H3	120.6
Cl1^i^—Au2—Cl2	90.05 (14)	C2—C3—H3	120.6
Cl1—Au2—Cl2	89.95 (14)	C3—C4—C5	121.4 (12)
Cl1^i^—Au2—Cl2^i^	89.95 (14)	C3—C4—H4	119.3
Cl1—Au2—Cl2^i^	90.05 (14)	C5—C4—H4	119.3
Cl2—Au2—Cl2^i^	180.00 (17)	C4—C5—C6	119.0 (12)
C2—N1—C6	119.6 (9)	C4—C5—H5	120.5
C2—N1—Au1	120.7 (8)	C6—C5—H5	120.5
C6—N1—Au1	119.7 (7)	N1—C6—C5	120.3 (11)
C2—C1—H1A	109.5	N1—C6—C7	117.4 (10)
C2—C1—H1B	109.5	C5—C6—C7	122.3 (11)
H1A—C1—H1B	109.5	C6—C7—H7A	109.5
N1—C2—C3	120.9 (12)	C6—C7—H7B	109.5
N1—C2—C1	118.2 (10)	H7A—C7—H7B	109.5
C3—C2—C1	120.9 (11)		
			
C6—N1—C2—C3	0.000 (4)	C3—C4—C5—C6	0.000 (5)
Au1—N1—C2—C3	180.000 (4)	C2—N1—C6—C5	0.000 (4)
C6—N1—C2—C1	180.000 (4)	Au1—N1—C6—C5	180.000 (3)
Au1—N1—C2—C1	0.000 (3)	C2—N1—C6—C7	180.000 (3)
N1—C2—C3—C4	0.000 (6)	Au1—N1—C6—C7	0.000 (3)
C1—C2—C3—C4	180.000 (5)	C4—C5—C6—N1	0.000 (4)
C2—C3—C4—C5	0.000 (6)	C4—C5—C6—C7	180.000 (4)

Symmetry codes: (i) −*x*, *y*, −*z*+1.

### Hydrogen-bond geometry (Å, °)

**Table:**

*D*—H···*A*	*D*—H	H···*A*	*D*···*A*	*D*—H···*A*
C3—H3···Cl1^ii^	0.93	2.77	3.572 (14)	145

Symmetry codes: (ii) −*x*+1/2, *y*+1/2, −*z*+2.
